# Lithium carbonate as add-on therapy to radioiodine in the treatment on hyperthyroidism: a systematic review and meta-analysis

**DOI:** 10.1186/s12902-021-00729-2

**Published:** 2021-04-12

**Authors:** Mohamed Abd-ElGawad, Mohamed Abdelmonem, Ahmed Eissa Ahmed, Omar Magdy Mohammed, Mohamed Sayed Zaazouee, Ahmed Assar, Mohamed Gadelkarim, Ahmed M. Afifi

**Affiliations:** 1 Faculty of Medicine, Fayoum University, 5 Al-Touba Street, from Al-Fanya Street, Al-Hadka road, Fayoum, Fayoum, Egypt; 2 Faculty of Medicine, Al-Azhar University, Assiut, Egypt; 3 Faculty of Medicine, Menofia University, Shebin El-Kom, Menofia Egypt; 4 Faculty of Medicine, Alexandria University, Alexandria, Egypt; 5 Department of Internal Medicine and Division of Digestive Diseases, College of Medicine, University of Kentucky, Lexington, USA

**Keywords:** Hyperthyroidism, Lithium carbonate, Radioactive iodine, Cure rate

## Abstract

**Background:**

The main purpose is to investigate the effect of LiCO3 as an add-on therapy with radioactive iodine in increasing the cure and decreasing the T4 level compared to radioactive iodine alone. The primary outcome is the cure rate as defined by the number of hyperthyroid patients who became euthyroid or hypothyroid. The secondary outcome is the T4 level.

**Methods:**

Four databases were searched (PubMed, Scopus, Web of Science, and Cochrane central library). The inclusion criteria were randomized and non-randomized clinical trials of hyperthyroidism patients receiving LiCO3 with radioiodine compared with hyperthyroidism patients receiving radioactive iodine alone. Included studies were appraised with the risk of bias version 2 tool, according to the Cochrane Handbook for Systematic Reviews of Interventions 5.1.0.

**Results:**

Nine studies were eligible for inclusion in the study, six randomized control trials and three non-randomized control trials. There were 477 patients in the intervention group and 451 patients in the control group. The cure rate was not significantly different between the two groups, while it was significantly increased with 5000 to 6500 mg optimized cumulative dose of LiCO3 compared with the control group*, P* = 0.0001. The T4 level showed no significant difference between the two groups, *P* = 0.13.

**Conclusions:**

LiCO3 adjunct to radioactive iodine did not show significant differences compared with radioactive iodine alone in terms of cure rate or decreasing T4 level. However, the dose of 5000 to 6000 mg of LiCO3 may increase the cure rate.

**Supplementary Information:**

The online version contains supplementary material available at 10.1186/s12902-021-00729-2.

## Background

Hyperthyroidism is a syndrome in which the thyroid gland is secreting large amounts of thyroid hormones as thyroxin (T4) and triiodothyronine (T3) [1]. It has some causes as Graves’ disease which is an autoimmune disease characterized by the presence of the anti-thyroid stimulating hormone (TSH), receptor antibodies with overproduction of T3 and T4 [2, 3], autonomous nodule or nodules with overproduction of thyroid hormones, some forms of thyroiditis with damage of thyroid follicles which resulted in the irregular release of T3 and T4, thyroid tumor or toxic goiter [1].

An initial approach to the treatment of hyperthyroidism is by administrating anti-thyroid drugs and aiming to reduce the production of thyroid hormones [4]. However, population-based studies [5] showed that anti-thyroid drugs might be associated with sudden cardiac death (3.9%). Besides, they had rare serious side effects as agranulocytosis, vasculitis, or hepatic injury which were considered as signs to stop these drugs [1]. The literature reported other less serious side effects as fever, pruritus, rash, arthralgia, gastrointestinal distress, and abnormal taste sensation [6]. Besides, this treatment is likely to fail after 18 months, and the recurrence of hyperthyroidism ensues [7]. Thus, nowadays, recommended treatments include radioiodine (RAI) therapy or thyroidectomy [4, 8].

Physicians at the Massachusetts General Hospital in Boston have used radioiodine in the treatment of thyrotoxicosis since 1941 [9, 10]. Owing to its convenient eight-day half-life and being an effective treatment of hyperthyroidism, it spread worldwide. Also, its risk of developing malignancy or even the mortality risk were not significantly elevated [11]. However, it had disadvantages as worsening exophthalmos in Graves’ disease patients with moderate or severe exophthalmos [12]. Also, it was advisable to be prevented in pregnant women for fear of its teratogenicity on the fetuses [1]. Besides, it could cause a sudden rise in thyroid hormones concentrations as a result of post radioiodine thyroiditis which increased the risk of cardiovascular disorders [1, 13]. Therefore, several adjunct therapies were used with RAI to increase its effectiveness, to prevent this acute increase of thyroid hormones, and to decrease its dose. One of these adjuncts was Lithium carbonate (LiCO3) [14].

Lithium salts were observed causing sedation in Guinea pigs, and LiCO3 in 1949 was introduced to patients with bipolar psychoses as a mode stabilizer [15]. The reason that lithium salts may be a suitable add-on with RAI is its significant inhibitory effect on the discharge rate of RAI from the gland [16], together with its ability to block the release of TH [17]. Lithium is also known to play a role in RAI retention in the gland being involved in blocking organic iodine and thyroid hormone release without effect on RAI uptake [18–20].

A cohort study in patients with Graves’ disease confirmed the above mentioned theory and showed a higher cure rate in patients treated with RAI with LiCO3, as add-on therapy, after one year [20]. This higher cure rate suggests that using lithium, as an adjunct to RAI therapy, in thyrotoxicosis can be useful. However, there are discrepancies in outcomes of using LiCO3 as adjuvant therapy with I131 among published studies [14, 21, 22]. We aim to solve these differences by performing a systematic review and meta-analysis to determine the overall effect of adding lithium carbonate to RAI in the treatment of hyperthyroidism.

## Methods

### Study design and registration

We followed the Preferred Reporting Items for Systematic Reviews and Meta-Analyses (PRISMA) statement [23] to conduct this systematic review (SR) and meta-analysis (MA) for clinical trials whether randomized (RCT) or non-randomized. The study has no registered online protocol.

### Inclusion criteria

We included studies that included patients with hyperthyroidism, Graves’ disease, or toxic goiter, which uses LiCO3 with I131 as an intervention and RAI only as a comparative. The study design included clinical trials, whether randomized or non-randomized. Only English-written human-based studies that provide published accessible full text were included.

### Primary outcomes

The primary outcome of the study was the cure rate of the patients, which is the number of euthyroid or hypothyroid patients after the treatment period.

### Secondary outcomes

The secondary outcome was the change in serum levels of total tetra-iodothyronine (T4) (ng/ml).

### Search strategy and study selection

We searched the PubMed, Scopus, Cochrane Central Register of Controlled Trials (CENTRAL) and Web of Science Core Collection databases from database inception through 27 July 2019. The search employed all relevant index terms and keywords and did not utilize any filters. The complete search strategy for all databases is available in the supplementary file.

The study selection process was done in two phases: title and abstract screening and full-text screening. Two independent investigators screened each item, and another investigator solved the conflict.

### Data extraction

We extracted the following data: the general features of the included studies, baseline characteristics, and outcomes of interest. General features included study design, country, timing, description of the intervention group, description of the control group, inclusion criteria, antithyroid drugs, duration of lithium administration, the reason for hyperthyroidism, and description of the intervention. Baseline characteristics included age (years), the onset of hyperthyroidism (months), gender (frequency), mean thyroid volume (ml), and mean serum TSH (mIU/I). The extracted outcomes were serum’s total T4 (ng/ml), and the number of euthyroid and hypothyroid patients (frequency).

### Dealing with missing data

Some data were reported as mean and SE, so we used Revman 5.3 software to convert SE to SD. Besides, we calculated the mean difference for Serum total T4 outcome by subtracting the mean of baseline from that of Post-treatment value. In addition, we obtained the SD of the change from baseline using the method described by Foolman 1992 [24] and Abrams 2005 [25] for calculating the SD of the change from baseline using a decided on correlation coefficient. The correlation coefficient was decided to be zero as a conservative approach to yield the highest possible SD to avoid significant false results.

### Statistical analysis

The extracted data regarding the cure rate was pooled as risk ratios, and the data about serum total T4 was pooled as mean differences. Both with the corresponding 95% confidence intervals in a random-effects meta-analysis model using the Mantel-Haenszel equation. Student T-test and Chi-square test were used to test the significant differences in the pooled data between the intervention and the control groups. We used Revman 5.3 software to perform the statistical analysis. We interpreted the results in forest plots for better visualization of data. We identified heterogeneity using the Chi-square heterogeneity test. We solved heterogeneity among studies by performing the leave-one-out sensitivity analysis. If the heterogeneity persisted, we used subgroup analysis. We used the random-effect model for heterogeneous data.

### Quality assessment

For the randomized controlled trials, we used the Risk of Bias version 2 (ROB2) tool. We followed the Cochrane Handbook for Systematic Reviews of Interventions 5.1.0. in using the tool [26]. The tool consists of five domains that assess quality by answering signaling questions that help the authors determine the overall quality in each domain. The five domains include assessing the risk of bias in the randomization process, the risk of deviation from intended interventions, the risk of missing outcome data, the risk of bias in the measurement of the outcomes, and the risk in selecting the reported results. For the non-randomized trials, we used the ROBINS-1 tool [27]. The tool assessed the studies through signaling questions to determine the risk of bias in seven different domains. The tool included the same four domains as ROB-2 except for the randomization bias, in addition to three other domains regarding the confounding bias, selection bias, and bias in the classification of interventions. Funnel plot were used to assess the risk of bias across the studies.

## Results

### Data collection and study selection

**Fig. 1:**
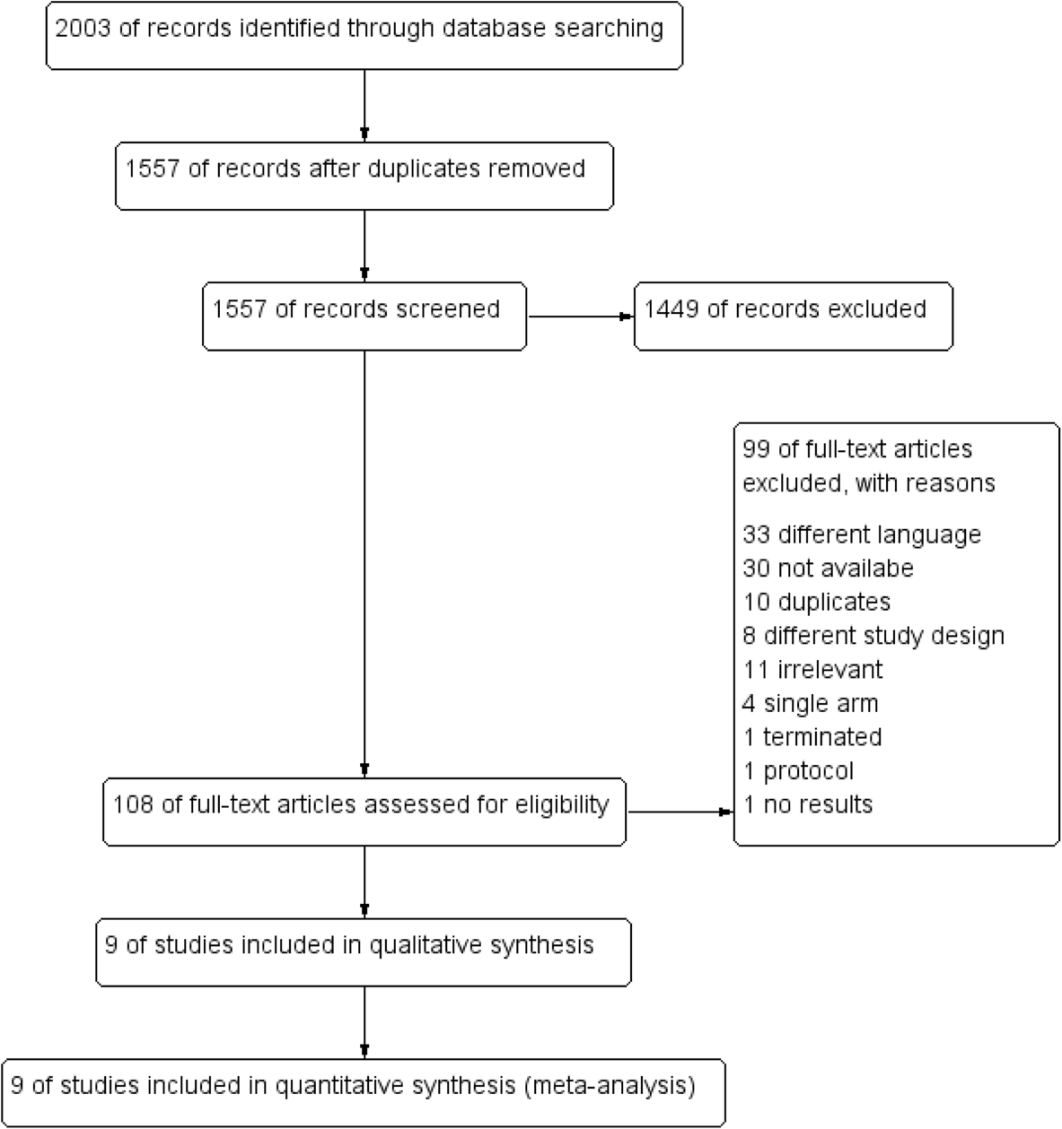
Flow chart of the study selection process

Data collection retrieved 2003 results; 446 of them were duplicates and removed. The remaining 1557 studies were involved in the title and abstract screening phase. Only 108 studies were eligible for the inclusion criteria and entered the full text-screening phase. Nine studies were included in qualitative synthesis and quantitative synthesis (meta-analysis) [14, 21, 22, 28–33]. Six were randomized controlled trials [14, 21, 22, 28, 29, 31] and three were non-randomized controlled trials [30, 32, 33]. The study selection process was illustrated in Fig. 1.

The nine studies included 477 patients in the intervention group and 451 patients in the control group, with a total of 928 patients. The general features and the baseline characteristics of the nine included studies were organized in Tables 1 and 2, respectively.

**Table 1:** Summary of the included studies

Study ID	Study design, country, and timing	Criteria	Intervention group(s)	Control group	Antithyroid drugs	Reason of hyperthyroidism
**Randomized controlled trials**						
**Thamcharoenvipas et al. 2019**	RCT, Thailand, between April 2015 and June 2016	The inclusion criteria were patients with thyroid gland weight > 50 g and age > 18 years	*N* = 20 3.7 MBq/g thyroid RAI plus **600 mg/day** LiCO3 for seven days N = 20 5.55 MBq/g RAI plus **600 mg/day** LiCO3 for 7 days	N = 20 7.4 MBq/g thyroid RAI without LiCO3	Propylthiouracil or Methimazole. (Stopped seven days before treatment)	Graves’ disease
**Hammond et al. 2016**	RCT, South Africa, between February 2014 and September 2015	The inclusion criteria were patients with hyperthyroidism who have Graves’ disease or Plummer’s disease	*N* = 88 RAI plus lithium **800 mg/day** for seven days	*N* = 75 RAI alone	Neomercazole, in some patients. (Stopped at least 5–7 days before treatment)	Graves’ disease or Plummer’s disease (toxic multinodular goiter and toxic adenoma)
**Lingudu et al. 2014**	RCT, India, from February 2011 to January 2012	Inclusion criteria were patients with hyperthyroidism aged > 18 years, with mild or absent Graves’ ophthalmopathy	N = 20 RAI plus lithium **900 mg/day** in three divided doses for six days	N = 20 RAI alone	Stopped seven days before RAI	Graves’ disease
**Bal et al. 2002**	RCT, India, from Dec 1994 to Dec. 1999	Exclusion criteria were patients with severe Graves’ ophthalmopathy, previous treatment of hyperthyroidism with radioiodine or surgery, and those with contraindications to lithium treatment	*N* = 164 Radioactive iodine plus lithium carbonate **900 mg/day** for three weeks	*N* = 152 Radioactive iodine with no lithium	carbimazole (stopped 3–4 days before the radioiodine therapy)	Graves’ disease, autonomous functioning thyroid nodule (AFTN), or a toxic multinodular goiter (TMNG)
**Bogazzi et al. 2002**	RCT, Italy, During the year 1999–2000	Inclusion criteria were patients with hyperthyroidism (Graves’ disease), aged > 20 years	N = 12 RAI plus lithium **900 mg/d** for 6 days *N* = 12 RAI plus lithium **900 mg/d** for 19 days	N = 12 RAI only	Methimazole (Stopped five days before RAI therapy)	Graves’ disease
**Bogazzi et al. 1999**	RCT, Italy, During the period 1994–1996	Inclusion criteria were patients with hyperthyroidism (Graves’ disease), aged > 20 years	*N* = 54 RAI plus lithium **900 mg/day** for 6 days	*N* = 46 RAI only	Methimazole (stopped five days before RAI therapy)	Graves’ disease
**Non-Randomized controlled trials.**						
**Sekulić et al. 2017**	Non-RCT, Serbia, from April 2012 to March 2016	The inclusion criteria were patients aged 20–70 years, with the gland size estimated by palpation as a grade 0 (normal-sized, invisible), grade 1 (slightly enlarged, visible), and grade 2 (moderately enlarged, highly visible)	N = 30 131I and LiCO3 **900 mg/day** for seven days	*N* = 30 131I alone	Stopped seven days before RAI	Patients with recurrent and long-lasting Grave’s hyperthyroidism
**Oszukowska et al. 2010**	Non-RCT, Poland, 2010.	The reported retrospective study involved 200 patients with hyperthyroidism, treated with radioactive iodine	N = 40 Radioiodine therapy plus lithium carbonate **750 mg/day** for ten days	*N* = 40 Radioiodine only	Not reported	Graves’ disease or toxic nodular goiter
**TURNER et al. 1976**	Non-RCT New Zealand, 1976	Inclusion criteria were patients with Diffuse thyroid hyperplasia as assessed by thyroid scan.	N = 16 131I (5 mCi) and lithium carbonate **400 mg daily** for one week before and one week after 131I	N = 16 131I (5 mCi) without lithium therapy	Carbimazole or propylthiouracil; (stopped one week prior to the start of the radioiodine)	Diffuse thyroid hyperplasia

RCT: Randomized controlled trial. N: number. RAI: radioactive iodine therapy. MBg: megabecquerel. mCi: millicurie

**Table 2:** Baseline characters of the patients in the included studies

Study ID	Study groups	Age (years) Mean (SD)	Gender (males) Number, percentage	The onset of hyperthyroidism (years) Mean (SD)	Mean thyroid volume (ml) Mean (SD)	Mean serum TSH (mIU/l) Mean (SD)
**Randomized controlled trials**						
**Thamcharoenvipas et al 2019**	Intervention 1	30 (7.4)	3, 15%	21.3 (32.6)	82.6 (30.4)	.005 (.006)
	Intervention 2	34.3 (13)	1, 5%	30.3 (39.3)	87.1 (21.0)	.011 (.02)
	control	31.8 (12.2)	8, 40%	15.2 (19.6)	98.9 (36.0)	.003 (.004)
**Hammond et al. 2016**	intervention	43.7 (13.2)	9, 10%	NA	NA	.05
	control	48.4 (12.0)	11, 14.7%	NA	NA	.04
**Lingudu et al. 2014**	intervention	35.9 (7.5)	2, 10%	19.7 (21)	26.7 (15.8)	NA
	control	37.3 (12.7)	9, 45%	15.5 (10.8)	28.2 (12.4)	NA
**Bal et al. 2002**	intervention	41.8 (12.2)	61, 37.2%	6.1 (47.9)	48 (29.0)	NA
	control	41.8 (11.5)	54, 35.5%	45.4 (41.6)	45 (24.0)	NA
**Bogazzi et al. 2002**	Intervention 1	48.0 (9.0)	2, 16.7%	37.3 (2.4)	19 (10)	1.3 (1.3)
	Intervention 2	51.0 (9.0)	3, 25%	5.7 (2.0)	21 (11.0)	1.4 (1.7)
	control	52.0 (13.0)	4, 33.4%	5.3 (1.8)	24 (13.0)	2.3 (1.8)
**Bogazzi et al. 1999**	Intervention	45 (12.8)	10, 18.5%	6.1 (2.3)	38 (22.0)	.6 (1.0)
	Control	51 (15.8)	9, 16.7%	5.7 (2.3)	35 (21.0)	.4 (.9)
**Non-randomized controlled trials**						
**Sekulić et al 2017**	Intervention	53.9 (8.8)	5, 16.7%	84.2 (68.0)	NA	.9 (.6)
	Control	51.3 (9.2)	6, 20%	72.6 (52.6)	NA	.9 (.88)
**Oszukowska et al 2010**	Intervention	52.1 (13.1)	29 (14.5%)	NA	NA	NA
	Control			NA	NA	NA
**TURNER et al 1976**	Intervention	44 (10.3)	5, 31.3%	NA	NA	NA
	Control	43 (7.8)	2, 12.5%	NA	NA	NA

Data is expressed as mean and standard deviation (SD) or frequency and percentage. NA: not availabe

### Quality assessment

Regarding random sequence generation, only two studies—Lingudu et al. [21] and Thamcharoenvipas et al. [31]—reported an appropriate method of randomization. However, the rest of the studies were judged as unclear due to insufficient information. Allocation concealment risk was unclear for all of the included studies due to insufficient information except for the Hammond et al. study [29]. It was judged as high-risk potential because the investigators could intervene with the patients’ assignment to the groups. The study of Thamcharoenvipas et al. [31] was judged as low-risk potential due to proper allocation concealment.

Regarding the blinding risk of bias, the Thamcharoenvipas et al. [31] study was a high-risk study concerning participants and personnel blinding because the physicians were not blinded. The study of Boggazzi et al., [14] was considered low-risk of bias regarding outcome assessors blinding because the examiners were sufficiently blinded. The rest of the included studies were considered as an unclear risk due to insufficient data.

Regarding the Incomplete outcome data bias, the studies of Bal et al. [22], Hammond et al. [29], and Boggazzi et al. 1998 [14] were considered as sources of high-risk bias due to their high patient attrition percentages (10, 12, and 9%, respectively), without any use of ITT method in the analysis of their data. The rest of the studies were judged as low risk.

**Fig. 2:**
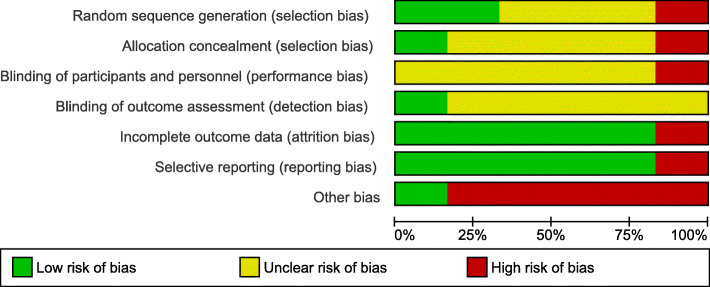
Risk of bias graph for randomized controlled trials using ROB2

**Fig. 3:**
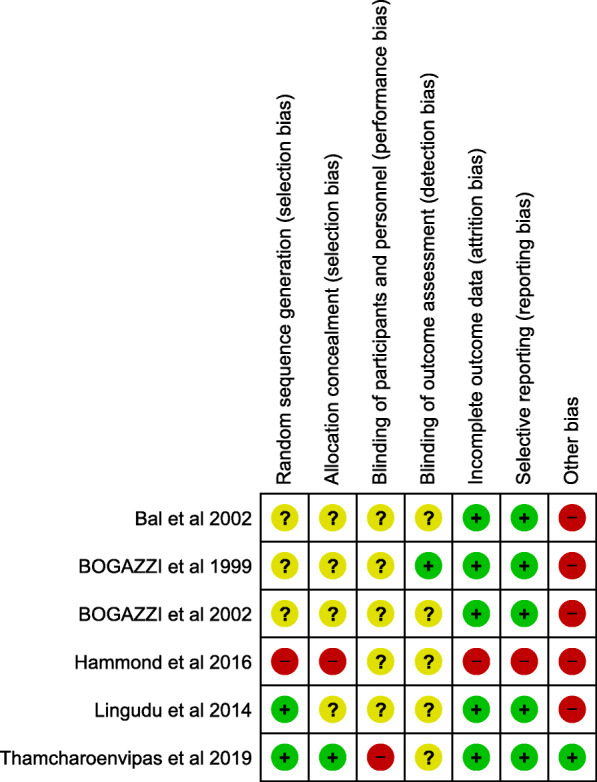
Risk of bias summary for randomized controlled trials using ROB2

For selective reporting, all studies were judged low risk except for the Hammond et al. [29] study. It was judged as high-risk potential due to incomplete reporting of the baseline characters of the patients’ biochemical data. Moreover, all included studies were considered as high-risk potential in the domain of other bias due to the unavailability of the study protocols. All the included studies ranged from unclear to moderate concerning overall quality (Figs. 2 and 3).

On the other hand, of the nonrandomized trials, two (Oszukowska et al. 2010 and Sekulić et al. 2017 studies) were judged as low-risk, while one (Brownlie et al. 1979 study) was judged as high-risk bias [30, 34, 35].

Both Oszukowska et al. 2010 and Sekulić et al. 2017 had low-risk in most the ROBINS-I domains; however, Oszukowska et al. 2010 study had some variability in measuring outcomes among all included patients (bias in the measurement of outcomes). The study of Brownlie et al. 1979 was considered high-risk because of lacking information in multiple domains including confounding and in the selection of participants into the study; moderate to high-risk were found among the domains of missing data, measurement of outcomes, and selection of the reported results (Table 3).

**Table 3:** Quality assessment of the nonrandomized trials using ROBINS-I

Study Domain	Oszukowska et al. 2010	Sekulić et al. 2017	Brownlie et al. 1979
**Pre-intervention**			
Bias due to confounding	Probably No	Probably No	No Information
Bias in selection of participants into the study	Probably No	No	No Information
**At intervention**			
Bias in classification of interventions	No	No	No
**Post-intervention**			
Bias due to deviations from intended interventions	No	No	No
Bias due to missing data	No Information	No Information	Probably Yes
Bias in measurement of outcomes	Probably Yes	No	Probably No
Bias in selection of the reported result	No	No	Probably Yes
**Overall risk of bias**	Low risk bias	Low risk of bias	High risk of bias

### Publication bias

**Fig. 4:**
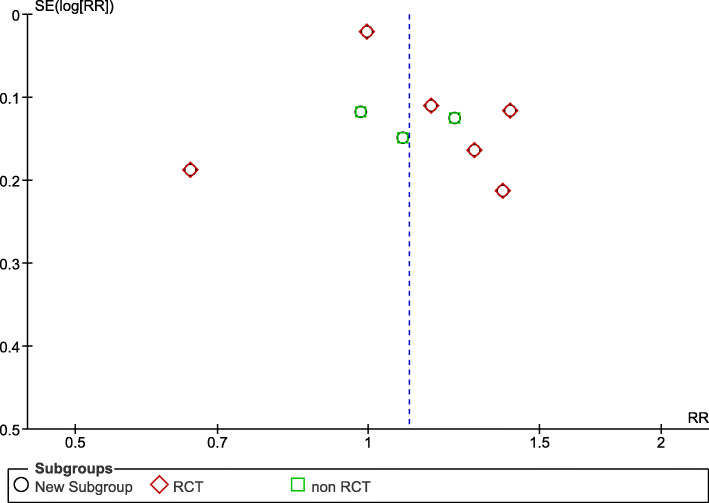
Funnel plot showing the risk of bias across the included studies

To assess the risk of bias across studies, a funnel plot was created for the main outcome. This analysis showed a significant risk of publication bias regarding the RCTs. On the other hand, the non-randomized controlled trials were nearly symmetrical in both sides of the middle line (Fig. 4).

### Primary outcomes

**Fig. 5:**
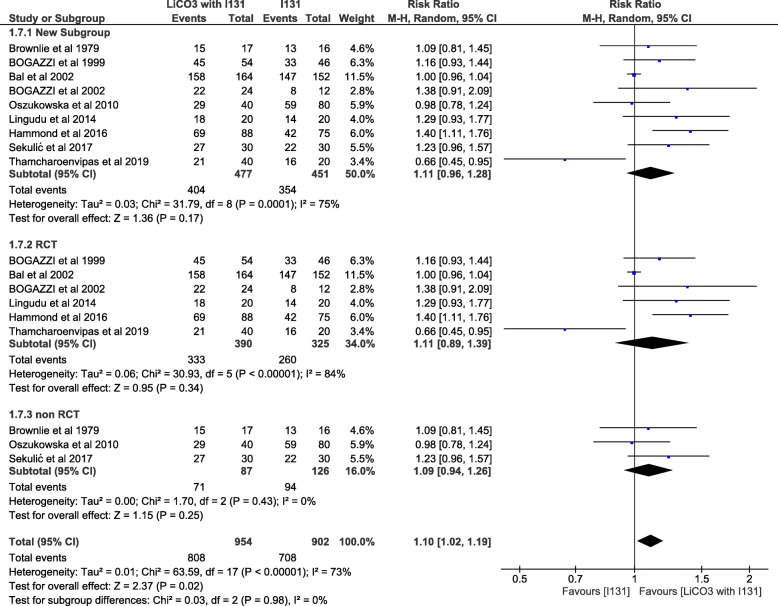
The cure rate defined as euthyroidism or hypothyroidism, sub grouped according study design whether randomized or not. LiCO3: lithium carbonate, I131: radioiodine 131

The total number of patients being treated in the Intervention group is 477 with a cure rate of 84.7% (404 patients), while the number being treated in the control group is 451 patients with a cure rate of 78.5% (354 patients), (RR = 1.11, 95% CI, .96–1.28; *P* = .17), not favoring any of the two compared groups. Substantial heterogeneity was inspected among the pooled studies (*P* = .0001, I^2^ = 75%) (Fig. 5).

The pooled data from the RCTs alone (6 studies) as a sub-group did not favor any of the two groups with a cure rate of 85.4% in the intervention group and 80% in the control group (RR = 1.11, 95% CI, .89–1.39; *P* = .34). Considerable heterogeneity was detected among the pooled studies (*P* = .0001; I^2^ = 84%) (Fig. 5).

Pooling the data of the non-RCTs (3 studies) as a separate sub-group also did not favor any of the compared groups with a cure rate of 81.6% in the intervention group and 74.6% in the control group (RR = 1.09, 95% CI, .94–1.26; *P* = .25). The pooled studies were homogenous (Fig. 5).

### Analysis of the studies according to the total dose

**Fig. 6:**
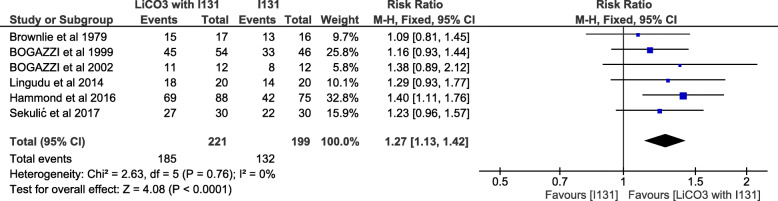
The cure rate defined as euthyroidism or hypothyroidism for a certain selected medium dose (5000–6500 mg) of lithium carbonate. LiCO3: lithium carbonate, I131: radioiodine 131

The overall cure rate of LiCO3 (optimized dose: 5000 to 6500 mg) significantly favored the Intervention (LiCO3) group over the control group—with the exclusion of extremely low [31] or extremely high [22, 33] doses of the study groups. The pooled results showed a cure rate of 83.7% in the intervention (221 patients) versus 66.3% in the control group (199 patients) (RR = 1.27, 95% CI, 1.13–1.42; *P* = .0001). The pooled studies were homogenous (*P* = .76, I^2^ = 0%) (Fig. 6).

### Secondary outcomes

**Fig. 7:**
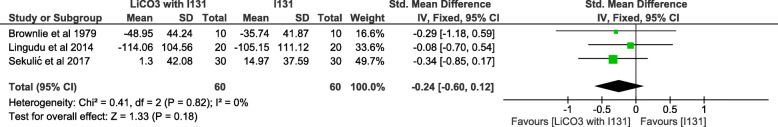
Serum total thyroxine (T4) as a secondary outcome. LiCO3: lithium carbonate, I131: radioiodin

The effect of the intervention on serum total T4 of the patients was reported in three studies (120 patients). The overall pooled result did not favor either of the two groups (SMD = -24.26, 95% CI, − 0.6 – 0.12; *P* = .18). The pooled studies were homogenous (*P* = .82, I^2^ = 0%) (Fig. 7).

## Discussion

This systematic review and meta-analysis study is based on nine clinical trials, including 928 patients with hyperthyroidism; 715 of them were randomly selected to RAI with lithium carbonate or RAI only. The rest of the studies were non-randomized. Our meta-analysis showed that the overall effect of LiCO3—5000 to 6500 mg for six or seven days—plus RAI led to better cure rates among hyperthyroidism patients than in the control group (RR = 1.24, 95% CI, 1.11–1.39). This result depends on the analysis of 420 patients in six trials [14, 21, 28–30, 32]. Conversely, the last meta-analysis reported non-significant differences in the cure rate between two groups of four randomized trials, including Bal et al., [22].

When we sub-grouped the included studies regarding their randomization, we noted that there was no significant difference regarding the cure rate between the intervention group and the control group of both randomized and non-randomized studies. These non-significant results may be due to the presence of trials with extremities in doses and durations. For instance, Oszukowska et al. [33] is a non-randomized trial in which the cumulative dose was 7500 mg for ten days. Moreover, Bal et al., [22] and Thamcharoenvipas et al., [31] are randomized trials in which the doses were 18,600 mg for 21 days and 4200 mg for seven days, respectively.

LiCO3 is administrated orally, totally absorbed, not metabolized, excreted unchanged by the renal system, and has a 12-h half-life. All of the dose is distributed through the body fluid, interstitial fluid, and eventually, slowly enters the cells [36]. RAI worsened the cure rate status in participants with a large thyroid gland, quick iodine washout, and diminished iodine uptake [37]. Lithium increased the half-life of RAI [16] and prevented the discharge of TH [17]. However, Thamcharoenvipas et al. [31] reported LiCO3 was harmful in rapid turnover Graves’ disease. This might be due to the whole dose of LiCO3 being 18,600 mg for three weeks which is considered as a high dose. Moreover, using LiCO3 with 3.7 mBq/g of RAI for Graves’ cases with rapid turnover uptake was a standard of use at their institution.

Regarding the secondary outcome, three studies disclosed decreased serum T4 without meaningful statistical variation between both the LiCO3 group and the control group. Sekulic et al. [30] showed an increase in T4 level and a decrease of TSH level for seven days in patients treated with RAI only. This reflex resulted from gland irradiation, while this phenomenon was lost in the LiCO3 group. Lingudu et al. [21] explicated that at four months, the group treated with RAI had undergone an abrupt drop of T4 and T3 levels. Of note, this rapid control may be helpful in elderly and cardiac patients.

The quality of the current evidence was considered good due to rigorous adherence to the Cochrane Handbook about systematic reviews and the PRISMA checklist in all the steps performed. Furthermore, we included nine trials in nine; six of them were randomized.

The strengths of our study can be summarized as we included only clinical trials that considered first-class evidence and applied multiple scales to appraise the same result. We did subgroup analysis according to randomization. In studies that did not report the correlation coefficient, we assumed it is zero as a conservative approach to yield the highest standard deviation and to avoid significant false results.

The limitations were that we included trials published in the English language only. We obtained the outcome data of Turner et al. from Brownlie et al. [32, 38]. Only nine controlled trials with different LiCO3 dosage regimens (six randomized and three non-randomized) matched our criteria of inclusion, six of them only included the preferred dosage. The most frequent dose used among studies exhibited significant results. In addition, the bias assessment of individual studies revealed an unclear risk of bias for several important domains, which indicates that a high degree of bias within these studies is possible. The risk of bias across studies revealed an asymmetrical publication pattern, indicating a potential bias in the body of literature. Taken together these indications warrant caution in the interpretation of the results. Further studies are needed to confirm these finding.

## Conclusion

This study suggests that the use of LiCO3 with a cumulative dose of 5000 to 6500 mg for approximately seven days, as adjuvant therapy to RAI, may be effective in hyperthyroid conditions, while the extremities doses of lithium may not be beneficial.

## Supplementary Information

**Additional file 1.**

## Data Availability

All data generated or analyzed during this study are included in this published article or in the data repositories listed in References.
