# Supplementary material for: Lithium carbonate as add-on therapy to radioiodine in the treatment on hyperthyroidism: a systematic review and meta-analysis
Source: BMC Endocr Disord. 2021 Apr 12;21:64. doi: 10.1186/s12902-021-00729-2 (PMC8040242; doi:10.1186/s12902-021-00729-2)
Supplement: Supplementary file 1 — Additional file 1. [file 12902_2021_729_MOESM1_ESM.docx]

**Lithium carbonate as add-on therapy to radioiodine in the treatment on hyperthyroidism: A systematic review and meta-analysis**

**Mohamed Abd-ElGawad^1*^, Mohamed Abdelmonem^2^, Ahmed Eissa Ahmed^3^, Omar Magdy Mohammed^4^, Mohamed Sayed Zaazouee^5^, Ahmed Assar^6^, Mohamed Gadelkarim^7^, Ahmed M Afifi ^8^**

1. *Medical student, Faculty of Medicine, Fayoum University, Fayoum, Egypt.*[*mohammed.mahmod87@gmail.com*](mailto:mohammed.mahmod87@gmail.com)
2. *Medical student, Faculty of Medicine, Fayoum University, Fayoum, Egypt.*[*mohamed.monem695@gmail.com*](mailto:mohamed.monem695@gmail.com)
3. *Medical student, Faculty of Medicine, Fayoum University, Fayoum, Egypt.*[*ai1340@fayoum.edu.eg*](mailto:ai1340@fayoum.edu.eg)
4. *Medical student, Faculty of Medicine, Fayoum University, Fayoum, Egypt.*[*om1305@fayoum.edu.eg*](mailto:om1305@fayoum.edu.eg)
5. *Medical student, Faculty of Medicine, Al-Azhar University, Assiut, Egypt.*[*mohamedzaazouee@gmail.com*](mailto:mohamedzaazouee@gmail.com)
6. *Medical student, Faculty of Medicine, Menofia University, Shebin El-Kom, Menofia, Egypt.*[*Ahmed.assar52@gmail.com*](mailto:Ahmed.assar52@gmail.com)
7. *Medical student, Faculty of Medicine, Alexandria University, Alexandria, Egypt.*[*m_abdelaati101070@alexmed.edu.eg*](mailto:m_abdelaati101070@alexmed.edu.eg)
8. *Clinical research fellow, Department of Internal Medicine and Division of Digestive Diseases, College of Medicine, University of Kentucky, Lexington, United States.*[*ahmad.abdelwhab@uky.edu*](mailto:ahmad.abdelwhab@uky.edu)

**Search strategy:-**

**PubMed:**

(hyperthyroidism OR graves disease OR graves' disease OR Thyroid Nodule OR toxic nodule OR Goiter, Nodular OR toxic multinodular goiter OR toxic multinodular goiter OR Basedow OR graves OR graves' OR toxic diffuse goiter OR goiter OR "Hyperthyroidism"(Mesh) OR "Graves Disease"(Mesh)) AND (LiCO3 OR Lithium Carbonate OR "Lithium Carbonate"(Mesh) OR lithium OR lithium compounds OR lithium compound)

**Cochrane:**

1. hyperthyroidism OR graves disease OR graves' disease OR Thyroid Nodule OR toxic nodule OR Goiter, Nodular OR toxic multinodular goiter OR toxic multi nodular goiter OR Basedow OR graves OR graves' OR toxic diffuse goiter OR goiter
2. LiCO3 OR Lithium Carbonate OR lithium OR lithium compounds OR lithium compound
3. #1 AND #2

**Scopus:**

ALL(hyperthyroidism OR “graves disease” OR “graves' disease” OR “Thyroid Nodule” OR “toxic nodule” OR “Goiter, Nodular” OR “toxic multinodular goiter” OR “toxic multinodular goiter” OR Basedow OR graves OR “graves'” OR “toxic diffuse goiter” OR goiter)) AND ALL(LiCO3 OR “Lithium Carbonate” OR lithium OR “lithium compounds” OR “lithium compound”)

**Web of Science:**

TS=(hyperthyroidism OR “graves disease” OR “graves' disease” OR “Thyroid Nodule” OR “toxic nodule” OR “Goiter, Nodular” OR “toxic multinodular goiter” OR “toxic multinodular goiter” OR Basedow OR graves OR “graves'” OR “toxic diffuse goiter” OR goiter)) AND TS=(LiCO3 OR “Lithium Carbonate” OR lithium OR “lithium compounds” OR “lithium compound”)

**Web of Science core collection:**

Science Citation Index Expanded (SCI-EXPANDED) --1900-present

Social Sciences Citation Index (SSCI) --1900-present

Arts & Humanities Citation Index (A&HCI) --1975-present

Conference Proceedings Citation Index- Science (CPCI-S) --1990-present

Conference Proceedings Citation Index- Social Science & Humanities (CPCI-SSH) --1990-present

Book Citation Index– Science (BKCI-S) --2005-present

Book Citation Index– Social Sciences & Humanities (BKCI-SSH) --2005-present

Emerging Sources Citation Index (ESCI) --2005-present
